# Nucleic Acid Aptamers Emerging as Modulators of G-Protein-Coupled Receptors: Challenge to Difficult Cell Surface Proteins

**DOI:** 10.3390/cells11111825

**Published:** 2022-06-02

**Authors:** Masaki Takahashi

**Affiliations:** Project Division of RNA Medical Science, The Institute of Medical Science, The University of Tokyo, Minato-ku, Tokyo 108-8639, Japan; tmasaki@ims.u-tokyo.ac.jp; Tel.: +81-3-5449-5324

**Keywords:** RNA aptamer, GPCR, modulator, SELEX

## Abstract

G-protein-coupled receptors (GPCRs), among various cell surface proteins, are essential targets in the fields of basic science and drug discovery. The discovery and development of modulators for the receptors have provided deep insights into the mechanism of action of receptors and have led to a new therapeutic option for human diseases. Although various modulators against GPCRs have been developed to date, the identification of new modulators for GPCRs remains a challenge due to several technical problems and limitations. To overcome this situation, a variety of strategies have been developed by several modalities, including nucleic acid aptamers, which are emerging as unique molecules isolated by a repetitive selection process against various types of targets from an enormous combinatorial library. This review summarized the achievements in the development of aptamers targeting GPCRs, and discussed their isolation methods and the diverse functional features of aptamers against GPCRs.

## 1. Introduction

Nucleic acid aptamers are single-stranded nucleic acids capable of specifically binding to targets [1,2,3], which widely range from chemicals to viruses, as well as cells based on shape complementarity [4]. To date, various aptamers have been used as sensing probes [5] and medical agents [6]. The first conceptual method of isolating aptamers, referred to as Systematic Evolution of Ligands by Exponential enrichment (SELEX), was established in 1990 [1,2,3]. Briefly, it was an iterative operation of selection and amplification, which allowed aptamers to be enriched in vitro from combinatorial libraries composed of approximately a quadrillion molecules that gave rise to a vast set of three-dimensional (3D) structures based on their primary sequence and chemistry. Unlike chemicals and antibodies, aptamers can achieve high specificity and affinity for their targets through multi-point bindings, such as hydrogen bonds, stacking, and van der Waals forces, across their relatively large contact area with the target [4].

To date, various aptamers have been developed, and more than 16,000 papers on aptamers have been reported in PubMed. Despite the increasing number of aptamer-related publications, only a few reports exist on aptamers targeting cell surface proteins, particularly in multi-pass transmembrane proteins, such as G-protein-coupled receptors (GPCRs). To the best of our knowledge, only five papers on aptamers targeting GPCRs have been reported so far, which is less than 0.1% of all papers related to aptamers. Molecules regulating GPCRs are vital and in high demand in the fields of basic science and drug discovery [7]; however, some technical challenges have limited their widespread application. In the first half of this review, we have introduced five successful cases of isolated aptamers targeting GPCRs. In the latter half, we have summarized features (pros and cons) of each GPCR-stabilizing material for SELEX and discussed the potential of functional aptamers as GPCR modulators along with pending issues and future perspectives from various viewpoints.

## 2. Successful Cases of Development of Aptamers Targeting GPCRs

**Figure 1:**
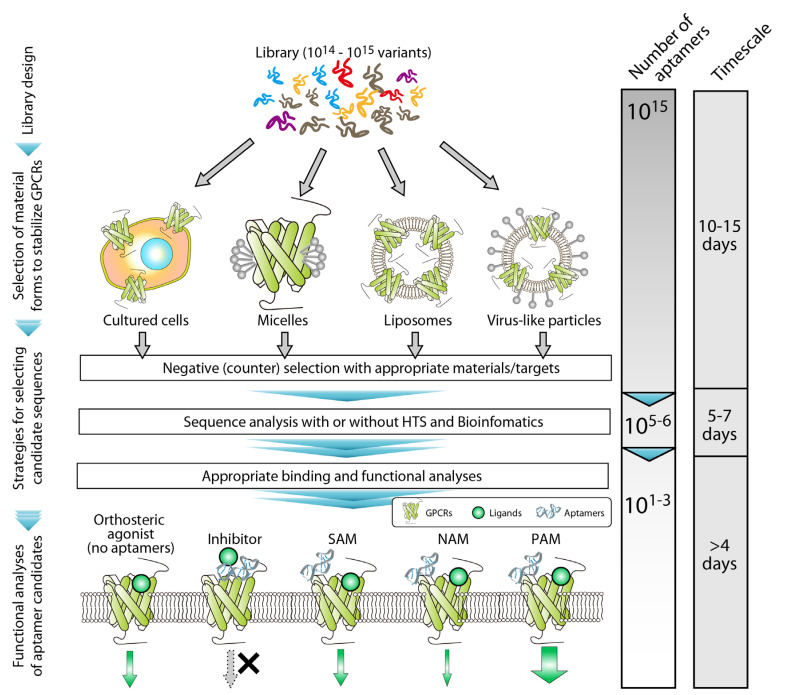
SELEX targeting GPCRs. Several studies successfully identified aptamers targeting GPCRs by using various selection materials and strategies of sequence analyses. The previous studies showed aptamers acting as inhibitors, silent allosteric modulators (SAMs), negative allosteric modulators (NAMs), and positive allosteric modulator (PAM) agonists. The expected change (decrease) in the number of sequences during SELEX and the average time required for each process are shown on the right side of the schema.

**Figure 2:**
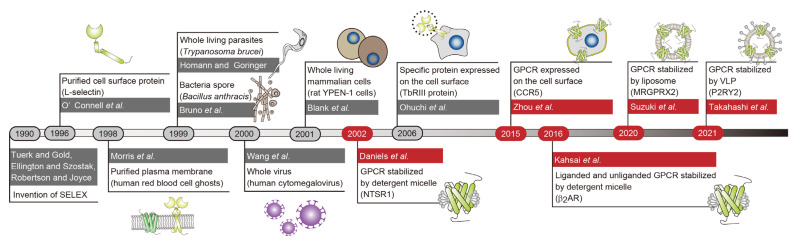
Development of iconic SELEX methods targeting GPCRs and other cell surface proteins. The year in which aptamers targeting GPCRs were reported is shown in red.

SELEX and aptamers targeting GPCRs reported to date are summarized below (Figure 1 and Figure 2, and Table 1).

### 2.1. First Aptamer Targeting a GPCR, Rat Neurotensin Type I Receptor (NTSR1)

In 2002, Dion A Daniels et al. generated the first RNA aptamer targeting a GPCR, rat neurotensin receptor NTS-1 (NTSR1) [8]. Neurotensin is a 13-amino acid neuropeptide found in the central nervous system [13] and gastrointestinal tract [14]; its receptor has been well analyzed structurally [15,16,17,18,19] (all structures of the receptor available in PDB) and has recently been focused upon as a therapeutic target [20]. To generate aptamers against NTSR1, the receptor was expressed in bacteria as a recombinant protein, acting as common SELEX bait, and then it was solubilized by several types of detergents, such as n-dodecyl β-D-maltoside (LM), 3-([3-cholamidopropyl] dimethylammonio)-1-propanesulfonate (CHAPS), and cholesteryl hemisuccinate (CHS); this suggested that the protein probably formed micelles using detergent components. For the selection process, histidine-tagged NTSR1 in detergents (micelles) was immobilized on magnetic beads for positive selection, and counter selection was performed with histidine-tagged osteopontin proteins to remove histidine-bound aptamers. After SELEX, eight sequences were identified as aptamers, specific to rat NTSR1 with affinity in the nanomolar range or less; a representative aptamer was named P19. Filter-binding assays showed that the P19 aptamer binds to human NTSR1 as well as rat NTSR1, and cell-based analysis showed that the P19 aptamer does not inhibit binding of the endogenous ligand (neurotensin) to the receptor.

The result indicated that the P19 aptamer did not enter and/or cover the ligand-binding pocket, thereby implying the binding of a silent allosteric modulator (SAM) or non-functional molecules binding to a non-orthosteric site. The first RNA aptamer targeting GPCR was the first aptamer acting as a non-orthosteric binding molecule of GPCR. Given the SELEX technology at the time of the study, it was a new challenge in two ways: SELEX using proteins in micelles and SELEX targeting GPCR.

### 2.2. Aptamers against CCR5: Aptamer-siRNA Chimeras as Therapeutic Agents for Infection-Related Diseases

After the identification of aptamers against NTSR1 in 2002, high-throughput sequencing (HTS) technology has rapidly developed and spread, and has been used in various experiments, including SELEX. As the first report of SELEX targeting GPCR by employing HTS in cell SELEX [9] (Figure 2), Jiehua Zhou et al. successfully identified several aptamers. Among the candidates was a representative sequence, G-3 aptamer, bound to C-C chemokine receptor type 5 (CCR5). CCR5 is a GPCR expressed in T cells and macrophages, and serves as a coreceptor for macrophage-tropic HIV-1 [21]. The G-3 aptamer showed binding affinity to CCR5-expressing cells with a Kd value of ~100 nM, and acted as an infection inhibitor with an IC50 value in the nanomolar range (50–350 nM). Based on the aptamer, the authors further designed and developed aptamer-siRNA chimeric nucleic acids that comprised a G-3 aptamer targeting CCR5 and siRNA targeting TNPO3, which is required for HIV-1 replication [22]. By using the aptamer as an infection inhibitor and as a delivery agent to HIV-1 susceptible cells, the chimeric molecules successfully inhibited HIV infection compared to the aptamer or siRNA alone.

This report suggested that aptamers targeting GPCRs can serve as protein–protein interaction (PPI) inhibitors, similarly to other common aptamers. Although several past studies had indicated that aptamer-siRNA chimeric molecules can possibly serve as HIV-1 therapeutic agents, their targets in aptamer parts were gp120 protein [23,24,25] and CD4 protein [26,27], both of which are not multi-pass transmembrane proteins. Thus, this study presented a valuable outcome to expand the range of target protein options to generate chimeric molecules with a delivery function.

### 2.3. Aptamers against A2a Adrenoceptor; Identification of Allosteric Inhibitors

In 2016, aptamers against β_2_-adrenoceptor (β_2_AR) were generated by Alem W. Kahsai et al., and this was the third report of aptamers against GPCRs [10]. The authors prepared β_2_AR from baculovirus-mediated expression in *Spodoptera frugiperda* (Sf9) insect cells and stabilized them in micelles, such as in the case of NTSR1 in 2002. As a unique and advanced point in this study, both liganded (BI-167107-bound) [28] and unliganded β_2_AR in micelles were subjected to SELEX to achieve selective isolation of aptamers binding to active or inactive receptors. For the efficient isolation of aptamers, HTS and bioinformatics tools were used in sequence analysis (Table 1). After screening for binding sequences based on the pull-down assay and biolayer interferometry (BLI) method, the authors focused on four aptamers, three out of which selectively bound to active (liganded) β_2_AR (named A1, A2, and A13 aptamers) and the fourth bound to inactive (unliganded) β_2_AR (named A16 aptamer). These aptamers possessed high affinity to each target in the nanomolar range, and stabilized the receptor conformations. Further analyses revealed that three out of the four aptamers binding to active β_2_AR showed inhibitory potency against the receptor in the micromolar range. In addition, electron microscopy (EM) imaging analysis indicated that inhibitory aptamers bound to the intracellular region of the receptor. Thus, a series of analyses suggested that the aptamers were negative allosteric modulators bound to the intracellular region of β_2_AR.

The study showed that the strategy to isolate aptamers selectively binding to active and inactive receptors can be achieved by preparing appropriate selection materials, such as liganded and unliganded β_2_AR. Regardless of receptor types, the screening method for aptamers with conformational preference was unique and important in aptamer development.

### 2.4. Aptamers against MRGPRX2; Inhibitor for Histamine Release from Mast Cells

Until 2020, recombinant GPCR proteins stabilized in micelles and cultured cells over-expressing GPCRs of interest have been used as selection materials for SELEX (Figure 2). Y Suzuki et al. established a new SELEX method targeting GPCRs by employing proteoliposomes, named “Proteoliposome-SELEX” [11,29]. Briefly, the authors developed aptamers against MRGPRX2, which is a crucial GPCR in non-IgE-dependent histamine release [30], by using liposomes to stabilize the GPCR produced from the wheat germ cell-free expression system. In the formation of the lipid bilayer, the authors used azolectin glycerosomes that contain a high concentration of glycerol in lipid particles to ensure the native fold of the GPCR [31]. For the selection of DNA aptamers, negative (counter) selection was conducted with mock liposomes lacking GPCR, and then positive selection was carried out with MRGPRX2-liposomes. The separation of bound and unbound sequences to the proteoliposomes was achieved by centrifugation (15,000× g). Based on the selection materials and separation process, a common SELEX process for DNA aptamers was carried out over 20 rounds, and successfully identified several aptamers against MRGPRX2. After screening, the authors confirmed that a representative aptamer, aptamer-X35, remarkably inhibits MRGPRX2-associated histamine release from mast cells in vitro.

The study demonstrated the utility of a new option, proteoliposome, in SELEX targeting GPCRs and other membrane proteins.

### 2.5. Aptamers against Purinergic Receptor P2Y2; Identification of PAM Agonist

**Figure 3:**
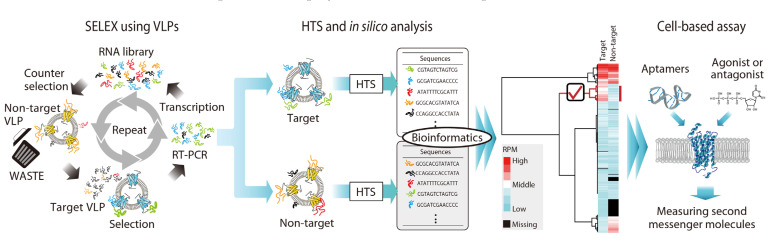
VLP-SELEX. The SELEX method using VLP, which was the most recently reported SELEX targeting GPCRs, was developed to isolate aptamers against P2RY2 in 2021. The method employed high-throughput sequencing (HTS) and bioinformatics tools in addition to VLP as a unique selection material for unstable cell surface proteins such as GPCRs.

In 2021, M Takahashi et al. established another SELEX method targeting GPCRs by employing virus-like particles (VLPs), referred to as VLP-SELEX (Figure 2 and Figure 3) [12]. VLPs are self-assembled viral nanoparticles without the viral genome, which is acceptable to express and display non-viral membrane proteins on their surfaces [32].

The authors employed VLP as a selection material for SELEX, which is expected to provide natural structure and orientation of GPCRs. In this method, VLP expressing target GPCR (human P2Y2 purinergic receptor, hP2RY2 [33]) and another isogenic VLP expressing non-target GPCR (human endothelin B receptor, hETBR) were prepared and used for selection and subtraction processes, respectively (Figure 3), similar to the strategy of the previously developed SELEX method “Icell-SELEX” [34]. To achieve effective screening, the non-target VLP (i.e., the VLP expressing non-target GPCR) was used not only for the subtraction process, but also for the estimation of false-positive sequences. After the selection process, an enriched library was mixed with target and non-target VLPs, and then sequences binding to each type of VLP were confirmed by HTS and analyzed by bioinformatics tools, such as FASTAptamer [35]. Finally, by comparing the read numbers of sequences that bound to target and non-target VLPs, sequences preferentially bound to the target VLPs were selected as potential sequences. For the separation of aptamers, ultrafiltration columns were used for isolation of the target-binding aptamers from free sequences based on the difference in their molecular sizes, thereby achieving an immobilization-free method to reduce the damage of VLPs and GPCRs. In this result, a representative sequence, named c37_8-40, was identified as a unique aptamer, with K_D_ values in the nanomolar range, which acts as a positive allosteric modulator depending on the binding state of the endogenous ligand.

Until this study, although some aptamers had been identified against GPCRs, their functions had been reported as PPI inhibitors and allosteric inhibitors, thereby indicating that they were all inhibitors. In terms of functions against GPCRs, this is the first report to show that the aptamers could act as PAM agonists for GPCRs.

## 3. Summary and Discussion on the Features of the GPCR Stabilization Materials for SELEX

The features of the four material forms reported to stabilize GPCRs—micelles, cultured cells, liposomes, and VLPs—are summarized below.

### 3.1. Features of Cultured Cells in SELEX

Among the material forms for SELEX targeting GPCRs, the most feasible and easily available are cultured cells, i.e., Cell-SELEX, as in the case of CCR5 in 2015 [9]. Cultured cells can express the native form of a membrane protein of interest, which possesses native post-translational modifications, such as glycosylation, disulfide bonds, and oligomerization, without specialized materials and techniques; thus, in terms of the availability and quality of GPCRs, cultured cells are superior to the other options as selection materials [36]. However, the development of aptamers is generally difficult to achieve, because the ratio of target GPCRs to non-target proteins is considerably low on the cell surface, and a few aptamers binding to target GPCRs in early SELEX rounds might be excluded until enrichment of the aptamers, due to entrapment with other non-target proteins in both positive and negative selection processes. Although various efficient Cell-SELEX methods have been developed to date [8,34,36,37,38,39,40,41,42], more improvements would be required for the achievement of easy-to-use Cell-SELEX targeting GPCRs.

### 3.2. Features of GPCRs Stabilized in Micelles for SELEX

In terms of the purity and the abundance of target GPCRs, detergent micelles are a promising material for SELEX targeting GPCRs. In fact, the micelles have been used for identifying aptamers against NTSR1 [8] and β2AR [10], thereby highlighting the utility of micelles in SELEX. However, it should be noted that the versatility of the micelle strategy does not seem to be certain at present, because GPCR stability is generally important for the structural analysis of GPCRs, and the structures of the above two GPCRs, NTSR1 and β2AR, have already been solved (Table 1) [16,43]; thus, these facts raise the possibility that micellar forms are adaptable for only a limited number of GPCRs with high structural stability, such as NTSR1 and β2AR. Further, GPCRs in micelles exposed both their extra- and intracellular regions, thereby allowing aptamers to bind to both regions. Thus, it would be essential to analyze whether aptamers isolated from this material bind to the intracellular or extracellular face of the GPCR [8,10]. Depending on the purpose of the study, this feature could be both an advantage and a disadvantage.

### 3.3. Features of GPCR-Embedded Liposomes for SELEX

Methods to generate proteoliposomes have been developed [44,45,46] and applied in cryo-EM analysis [47] and antibody screening [48]. Nowadays, proteoliposome generation methods have been diversified [49] and seem to be becoming a feasible option for the selection material of SELEX. Albeit just one case, aptamers targeting MRGPRX2 have been isolated by using proteoliposomes [11]. As a positive feature of liposomes, the abundance (purity) of target GPCRs is expected to be high, as in the case of micelles, thereby implying efficient aptamer selection. In contrast, the orientation of GPCRs on liposomes could not be regulated with the current technology. Therefore, similarly to the micelles, it is uncertain whether isolated aptamers binding to proteoliposomes are bound to the intra- or extracellular region. Besides, in general, liposomes take different sizes and forms (uni- and multilamellar, and multivesicular vesicles), depending on the method of production and various other conditions [50], which are expected to affect the efficiency of aptamer selection; hence, the quality control of this material seems to be more difficult than in micelles. Regarding the versatility of liposome as a selection material for GPCRs, given that the structure of MRGPRX2 has recently been solved by cryo-electron microscopy (Cryo-EM) in 2021 (Table 1) [51], there is the possibility that adaptable GPCRs for proteoliposomes are limited to structurally stable GPCRs only, as in the cases of NTSR1 and β2AR in micelles. Taken together, it seems that the features, as well as the pros and cons of using proteoliposomes, are totally similar to that in the case of micelles, and they can be expected to be a new feasible and promising option as selection materials for SELEX targeting relatively stable GPCRs, along with micelles.

### 3.4. Features of GPCR-Embedded VLPs in SELEX

To date, various VLPs have been generated and used in various fields, such as vaccine development, delivery systems, and antibody screening [32]. As a positive feature of VLPs as selection materials for SELEX, VLPs are easier to manipulate than cells. Further, unlike micelles and liposomes, they do not require difficult and delicate purification processes and can naturally achieve physiological orientation of the receptor. In addition, VLPs are generally considered to be cryopreservable (i.e., easy-to-use material compared to live cells), and there is no risk of membrane fusion, as in the case of liposomes. On the other hand, as for the negative features of VLPs, such as cultured cells, negative selection appears to be essential for the effective selection of VLPs, since non-target membrane proteins (e.g., VSVG) are displayed on their surface. In other words, both the feasibility and difficulty of the SELEX method using VLPs seem to be intermediate between live cells and micelles (liposomes), and, hence, VLPs might potentially become relatively convenient materials for SELEX targeting GPCRs.

## 4. Conclusions and Perspectives

As described in this review, aptamers targeting GPCRs have had few successes and there is no definitive method to identify them at present. However, the basis for the generation of GPCR-modulating aptamers is surely being established by the expansion of selection materials and the uncovering of the functional potential of aptamers for GPCRs as PPI inhibitors and/or receptor conformation regulators.

Previous successful cases, introduced herein, clearly emphasized the importance of GPCR-stabilizing materials and of the stability of target GPCRs in the development of aptamers targeting GPCRs, since all the GPCRs introduced in this review had been stabilized with some materials to mimic a native membrane environment, such as micelles, liposomes, VLPs, or actual cell membranes in cultured cells. There was no case of the usage of common naked recombinant proteins as selection materials, thereby emphasizing the importance of retaining the structure of GPCR and its supporting materials for aptamer development. Regarding the stability of GPCRs, needless to say, the stability of the GPCR itself is one of the key factors in aptamer discovery. Indeed, four out of five studies reporting GPCR aptamers had targeted NTSR1, β2AR, CCR5, and MRGPRX2, whose three-dimensional structures have already been solved, suggesting that there is a close relationship between GPCR stability and successful SELEX. In contrast, the structure of P2RY2 is yet to be solved; therefore, VLPs may be speculated to be suited as stabilization materials, even in unstable GPCRs. In future, GPCR-stabilizing materials for aptamer development might be expanded to nanodiscs [52], SMALP [53], and other new materials.

In addition to the diversification of selection materials for GPCRs, it would be noteworthy that aptamers targeting GPCRs have been demonstrated to exhibit a variety of functions, not just as PPI inhibitors. Aptamers targeting CCR5 and MRGPRX2 showed inhibitory activity against the target receptors, which might be based on the mechanism of protein–protein (ligand–receptor) interaction (PPI) inhibition as a major aptamer function. On the other hand, although the aptamer targeted NTSR1 bound to the target GPCR with nanomolar affinity, it allowed the interaction of the neurotensin ligand with the receptor. The authors reporting NTSR1 aptamers expected that aptamers bind to extracellular regions, except for the ligand-binding domain, considering the result from the binding assay using NTR-expressing CHO cells, thereby suggesting that aptamers are silent allosteric modulators (SAMs). Although characterization of the aptamer might not be fully accomplished, the aptamers appear to be first to bind to the allosteric site of GPCRs. In addition to this case, aptamers targeting β_2_AR were identified as negative allosteric modulators (NAMs) binding to the intracellular sites as per cyro-EM. Further, aptamers targeting P2RY2 acted as positive allosteric modulators (PAMs) and agonists, depending on the intrinsic ligand-binding state. The aptamers targeting P2RY2 were expected to bind to extracellular allosteric sites, since PAM activity was examined by cell-based assays, and SELEX was conducted by VLP displaying P2RY2, which was anticipated to be the native orientation on the membrane. Thus, the reports demonstrated the great potential of nucleic acid aptamers that could act as NAL, NAM, and PAM agonists, as well as simplistic PPI inhibitors for GPCRs, according to the specificity of aptamers and vast diversity of the RNA library.

Based on the successful cases described above, it seems that aptamers targeting GPCRs can be identified as allosteric modulators. One possible reason for this might be the relationship between the physical size and location of the orthosteric pocket in GPCRs. Ligand-binding sites or orthosteric sites of GPCRs classified as class A are generally located deeper than the allosteric sites [54]. Further, ligand-binding cavities were estimated to be 100–1200 (Å^3^) in volume [55], whereas the volume of aptamers was estimated to be more than 10,000 (Å^3^), even in relatively small (17 nucleotides) thrombin aptamers [56,57]. Thus, aptamers unlikely bind to the inside of orthosteric pockets, even if the aptamers can cover the entrance of the pocket. Given the molecular features of aptamers, aptamers generally interact with their target at multiple points over a large contact surface compared to small molecules or antibodies [4]. Such multipoint interactions may affect and limit conformational changes in or the activation of GPCRs, possibly leading to the isolation of allosteric-modulating aptamers. Conversely, this feature seems to be unsuitable for the isolation of orthosteric modulators in aptamers as a GPCR modulator.

In the cases of RNA aptamers targeting GPCRs, all of the aptamers possessed chemical modifications. The aptamers targeting NTSR1, Beta2AR, and P2RY2 have 2′-fluoro-pyrimidene as a major chemical modification in RNA aptamers, which is well known to confer RNase resistance [58]. In the case of aptamers targeting P2RY2, 2′-deoxy ATP was incorporated into the aptamers in addition to 2′-fluoro-pyrimidene. One might speculate that the modification of ribose in aptamers largely contributed not only to RNase resistance, but also affinity to targets. Further, several artificial bases, such as SOMAmer in 2010 [59], Ds-base in 2013 [60], and GACTZP-DNA in 2014 [61], have been developed and are considered to be useful for selecting high-affinity aptamers due to their hydrophobic interaction, which is a weakness of aptamers with natural bases. SOMAScan technology based on SOMAmer can target one GPCR, 5HT2A receptor, by epitope-based selection [62]. Although aptamers with artificial bases have no achievement in GPCR modulation at present, artificial bases possessing unique features might be a key player in generating GPCR aptamers in the future.

Pending issues related to aptamer technologies targeting GPCRs still remain. For example, confirmation of the intact structure and function of target GPCRs in micelles, liposomes, and VLPs is difficult. Therefore, there is no guarantee that aptamers indeed bind to GPCRs in an actual cell membrane, even though candidate sequences bind to GPCRs stabilized in artificial environments. Besides, there are only a few robust methods to detect molecular interactions between candidate sequences and GPCRs stabilized in each material. In fact, SPR analysis, commonly used as the first screening, did not always work well for micelles, liposomes, or VLPs in our experience (data not published). Flow cytometry and pull-down assays (or others) may be available, but lack the throughput capacity, limiting the chances of identifying promising sequences. Thus, improvement of such basic technologies for screening binding molecules would be key to the future development of aptamer technologies and the regulation of GPCRs.

In addition to the development of selection materials or others in wet analyses, HTS and computational science are essential for the efficient development of aptamers [63], particularly in difficult targets, such as GPCRs. The three out of five papers introduced here reportedly used HTS and/or bioinformatics tools [9,10,12]. Recently, there has been a notable trend toward the adoption of machine learning and artificial intelligence (AI) technologies for aptamer identification and aptamer design refinement [64,65,66,67]. Combined with appropriate data from wet analysis, such as counter selection and negative control to estimate false-positive sequences [12], aptamer identification is expected to become more efficient as a multidisciplinary technology. In future, the trend in aptamer drug development might shift from exploration to design, based on the various accumulating data, such as GPCR and aptamer structures.

Since this review is based on a few reports on aptamers targeting GPCRs, as a limitation of the present review, there is the potential risk of bias in comments and discussions on the aptamer development for GPCRs. To address this, further exploration and detailed analyses of aptamers targeting GPCRs are needed to gain a deeper insight into their mechanisms of action as GPCR modulators, in order to establish a definitive GPCR-aptamer generation and design strategy.

With advances in various fields related to GPCRs and aptamer technologies, GPCR-modulating aptamers may have a major beneficial impact on human health and basic science in the future.

## Figures and Tables

**Table 1:** Reports on aptamers targeting GPCRs.

Target GPCRs	3D Structures of GPCR (human)	Selection Material Forms	Expression System for GPCRs	Negative Selections	Separation	Sequencing Methods	Bioinformatics Tools	Property of Aptamers	DNA or RNA aptamers	Names and Sequences of Representative Aptamers	Impact of Aptamers on Each Target GPCR	Reference
NTSR1	Solved (PDB ID: 4GRV)	Micelles	*E.coli*	Histidine-tagged osteopontin (immobilized on the beads)	Paramagnetic beads	*E.coli*	None	SAM (or non-functional binding moelcules)	RNA (2’-fluoro-pyrimidine)	Name, P19; 5’-GGGAGGACGAUGCGGACAGAUACGGAACUACAGAGGUCAAUUACGGUGGCCACGCCAGACGACUCGCCCGA-3’	Not determined	[8]
CCR5	Solved (PDB ID: 4MBS etc.)	Cells (U373-Magi-CCR5E)	Human cancer cell lines (U373-Magi-CCR5E)	U373-Magi cells	Incubation and washing on culture dishes	HTS	Original analysis method	Inhibitor	DNA	Name, G-3; 5’-GGGAGGACGATGCGGGCCUUCGUUUGUUUCGUCCACAGACGACTCGCCCGA-3’	70% inhibition (HIV infection)	[9]
Beta2AR (liganded and unliganded)	Solved (PDB ID: 2R4S etc.)	Micelles	Baculovirus-mediated expression in Sf9 insect cells	AT1aR in micelle	Nitrocellulose	*E.coli* and HTS	CutAdapt, FASTX-Toolkit, Clustal Omega, Mfold	NAM and SAM (or non-functional binding moelcules)	RNA (2’-fluoro-pyrimidine)	Name, A13; 5’-GGGAGGACGAUGCGGGUCUUAGCUCUGCAGCCCACGGAGGAGAGGGGAGGGCCGACAGACGACUCGCUGAGGAUCCGAGA-3’	46% inhibition (isoproterenol-stimulated adenylyl cyclase activity)	[10]
MRGPRX2	Solved (PDB ID: 7S8N etc.)	Liposomes	Wheat germ cell-free protein expression system (WEPRO7240 Expression Kit)	Liposome lacking GPCR	Centrifugation	Not mentioned	Mfold	Inhibitor	DNA	Name, X35; 5’-ATGACCATGACCCTCCACACTGTAGGCACCACGGGTCCCTGGCAGTTAAAAGTACGTTTGTCAGACTGTGGCAGGGAAACA-3’	70% inhibition (histamine release)	[11]
P2RY2	Unsolved	VLPs	Expi293 cells (Expi293™ MembranePro™ Expression System)	ETBR in VLP	Ultrafiltration spin column	HTS	FASTAptamer, Clustral Omega, TreeView, Cluster, MEME, CentroidFold	PAM agonist	RNA (2’-fluoro-pyrimidine + deoxy-ATP)	Name, c37_8-40; 5’-ACCGUCGUACGAUGGUUUAACAUCGUGCAGACG-3’	20% activation [as agonist], 60% inhibition [as antagonist], 120–300% activation [as PAM] (calcium signaling)	[12]

## Data Availability

Not applicable.
